# Autoreactive B cells recruited to lungs by silica exposure contribute to local autoantibody production in autoimmune-prone BXSB and B cell receptor transgenic mice

**DOI:** 10.3389/fimmu.2022.933360

**Published:** 2022-08-02

**Authors:** Lanette Fee, Advika Kumar, Robert M. Tighe, Mary H. Foster

**Affiliations:** ^1^ Department of Medicine, Duke University Health System, Durham, NC, United States; ^2^ Medical Service, Durham Veterans Affairs (VA) Medical Center, Durham, NC, United States

**Keywords:** silica, autoimmunity, BXSB, autoantibody transgene, TLR

## Abstract

Occupational exposure to inhaled crystalline silica dust (cSiO2) is linked to systemic lupus erythematosus, rheumatoid arthritis, systemic sclerosis, and anti-neutrophil cytoplasmic autoantibody vasculitis. Each disease has a characteristic autoantibody profile used in diagnosis and implicated in pathogenesis. A role for cSiO2 in modulating humoral autoimmunity *in vivo* is supported by findings in mice, where respirable cSiO2 induces ectopic lymphoid structures as well as inflammation in exposed lungs across genetically diverse backgrounds. In lupus-prone mice cSiO2 exposure also leads to early onset autoantibody production and accelerated disease. Elevated autoantibody levels in bronchoalveolar lavage fluid (BALF) and lung transcriptome analysis suggest that the lung is a hub of cSiO2-evoked autoimmune activity. However, mechanisms by which cSiO2 and lung microenvironments interact to promote autoantibody production remain unclear. We previously demonstrated elevated anti-DNA Ig in BALF but not in lung cell cultures from cSiO2-exposed C57BL/6 mice, suggesting that BALF autoantibodies did not arise locally in this non-autoimmune strain. Autoantibodies were also elevated in BALF of cSiO2-exposed lupus-prone BXSB mice. In this report we test the hypothesis that dysregulated autoreactive B cells recruited to cSiO2-exposed lungs in the context of autoimmune predisposition contribute to local autoantibody production. We found that anti-DNA and anti-myeloperoxidase (MPO) Ig were significantly elevated in cultures of TLR ligand-stimulated lung cells from cSiO2-exposed BXSB mice. To further explore the impact of strain genetic susceptibility versus B cell intrinsic dysfunction on cSiO2-recruited B cell fate, we used an anti-basement membrane autoantibody transgenic (autoAb Tg) mouse line termed M7. In M7 mice, autoAb Tg B cells are aberrantly regulated and escape from tolerance on the C57BL/6 background. Exposure to cSiO2 elicited prominent pulmonary B cell and T cell aggregates and autoAb Tg Ig were readily detected in lung cell culture supernatants. Taken together, diverse disease-relevant autoreactive B cells, including cells specific for DNA, MPO, and basement membrane, are recruited to lung ectopic lymphoid aggregates in response to cSiO2 instillation. B cells that escape tolerance can contribute to local autoantibody production. Our demonstration of significantly enhanced autoantibody induction by TLR ligands further suggests that a coordinated environmental co-exposure can magnify autoimmune vulnerability.

## Introduction

Considerable evidence indicates that gene-environment interactions underlie susceptibility to autoimmunity. This disease spectrum has potentially devastating clinical consequences that affect approximately 5% of the US population (1). Systemic autoimmunity poses a particular threat because of its multi-organ involvement and chronic relapsing nature, as is typical for systemic lupus erythematosus (SLE), rheumatoid arthritis (RA), systemic sclerosis (SS), and anti-neutrophil cytoplasmic antibody (ANCA) vasculitis (AAV). A fundamental defect in these diseases is the perturbation in immune regulation that allows escape from normal recognition of self, resulting in activation of autoreactive lymphocytes. It is thus not surprising that variants in genes encoding immune-related proteins are prominent within susceptibility loci linked to autoimmunity (2). Many of these systemic autoimmune disorders, including SLE, RA, SS, and AAV, are also linked to occupational exposure to inhaled crystalline silica dust (cSiO2) (3, 4). Silicon dioxide is a principal component of the earth’s crust and is prevalent in silica-rich rocks and soil. Aerosolization of cSiO2 particles and exposure to respirable dust are common in farming, rock grinding, mining, blasting, and construction. Understanding how cSiO2 inhalation interacts with genetic susceptibility and modulates immune regulation will provide important insight into mechanisms underlying human autoimmunity and inform design of novel interventions for these debilitating disorders.

The mechanisms by which inhaled silica influences humoral immunity are of particular interest because of the key role autoantibodies play in the diagnosis and pathogenesis in autoimmune diseases. In SLE, a prototypical systemic autoimmune disease, major disease manifestations are attributed to deposition of autoantibodies or immune complexes in tissues, organs, or blood cell surfaces (5–7). SLE diagnosis relies on identification of serum autoantibodies and in some clinical settings autoantibody levels correlate with disease activity (8–10). Similarly, anti-citrullinated protein Ig, anti-topoisomerase Ig, and anti-myeloperoxidase (MPO) or anti-proteinase 3 autoantibodies are central to diagnosis and pathogenesis in RA, SS, and AAV, respectively (11–13). Antibody deposition may alter cell activation or function or lead to activation of complement and Fc receptors. In addition, agents specifically targeting B cells are among the immune modulators approved or under investigation to control disease (14–18).

In autoimmune-prone mice, pulmonary instillation of cSiO2, when compared to vehicle-instilled counterparts, increases levels of circulating autoantibodies. The specific composition of autoantibodies following cSiO2 can vary. While comparisons across studies are difficult due to differences in cSiO2 dosing, duration, mouse strain, sex, autoantibody specificity, and isotype, it is clear that cSiO2 elicits anti-nuclear autoantibodies, a hallmark of SLE. In certain strains with aggressive lupus (i.e. NZM2410, NZBWF1, and New Zealand Black, or NZB, lupus mice), cSiO2 exposure increases ANA, anti-histone, anti-dsDNA, anti-ssDNA, and/or anti-RNP, Sm, Ro/SSA, and La/SSB Ig (19–28). In cSiO2-exposed female NZBWF1 mice, plasma assayed by an autoantigen protein microarray also revealed autoantibodies reactive with a diverse assortment of cytoplasmic, extracellular, and matrix proteins (27, 28). Supporting pathogenic consequences from these autoantibodies, cSiO2-exposed NZM2410 and NZBWF1 mice exhibit accelerated autoimmune renal pathology, including increased kidney IgG and complement deposition, proteinuria, and histopathologic changes consistent with glomerulonephritis (20, 22–24, 26). Enhanced autoantibody production may not be limited to spontaneous disease models; in diversity outbred mice, cSiO2 exposure increases serum levels of ANA+ IgG reactive with ENA5, RNP, Sm and dsDNA (29). This supports the notion that cSiO2 exposure in mice broadly induces autoantibody production, though the mechanisms for this induction are not well understood.

Understanding how cSiO2 exposure modulates humoral immunity *in vivo* and the relative role of systemic versus local immune cSiO2 responses are topics of active investigation. In animals, cSiO2 respiratory instillation leads to well characterized acute and chronic inflammatory responses, as reviewed in (30). Infiltrating neutrophils, monocytes, and macrophages release proinflammatory and profibrotic cytokines such as TNFalpha, IL-1, and TGFbeta, and contribute to progressive lung fibrosis reminiscent of silicosis in humans (22, 26, 29, 31–33). This injury may be a causative factor in autoantibody generation as cycles of macrophage cell death and impaired apoptotic cell clearance increase availability of lupus autoantigens (34, 35). Lymphocytes are also recruited to cSiO2-damaged lungs and form ectopic lymphoid structures, a phenomenon observed across genetically diverse backgrounds, including non-autoimmune strains (22, 25, 29, 32, 33). In addition this may lead to activation of T cells and B cells in these structures, as it has been shown that cSiO2 exposure in NZBWF1 female mice upregulates lung expression of multiple genes engaged in lymphocyte activation and function, including Blnk, Cd19, Cd180, Tlr9, Cd80, Cd86, Fcgr2b, Tnfrsf4, and Ctla4, and leads to detectable B cell activating factor (BAFF) levels in BALF (36, 37). These changes are also associated with increased autoantibody levels in BALF. Anti-dsDNA Ig are significantly elevated in BALF of cSiO2-exposed NZBWF1 mice (23, 24, 26). Interestingly, these BALF IgG autoantibodies appear several weeks prior to their detection in plasma and IgG-producing plasma cells are detected in inflamed cSiO2-exposed lungs (22, 27, 28), suggesting local autoantibody production in lupus-prone NZBWF1 mice. We confirmed this possibility in autoimmune-prone MRL/MpJ mice, in which anti-DNA IgG levels are elevated not only in BALF from cSiO2-exposed mice but also in culture supernatants of stimulated lung cells (25).

In the current studies we directly test the hypothesis that anti-DNA and anti-MPO autoreactive B cells recruited to the lungs of cSiO2-exposed female lupus-prone BXSB mice are a source of autoantibodies. BXSB mice are genetically distinct from other common inbred lupus strains and are predisposed to develop autoimmune membranoproliferative glomerulonephritis (38). We show that anti-DNA and anti-MPO Ig are significantly elevated in culture supernatants from Toll-like receptor (TLR)-stimulated lung cells isolated from cSiO2-exposed BXSB mice. Consistent with the observations described by Pestka and colleagues in NZBWF1 mice (23, 27, 28), our findings suggest that genetically-determined preexisting immune dysregulation facilitates autoantibody production by lung-recruited B cells in the cSiO2-damaged microenvironment. To extend these observations and test the impact of preexisting B cell dysregulation in the context of a non-autoimmune genetic background, we used a unique mouse autoantibody (autoAb) transgene (Tg) reporter line termed M7 (39, 40). AutoAb Tg B cells in these mice express an Ig that binds to basement membrane laminin, an autoantibody target in several autoimmune diseases including SLE and a specificity detected in BALF and serum of NZBWF1 mice exposed to cSiO2 (27, 41, 42). M7 Tg B cells are aberrantly regulated and escape from tolerance on the C57BL/6 background (39, 40). We show that exposure of these autoAb Tg mice to instilled cSiO2 elicits prominent pulmonary lymphoid aggregates and recruits autoreactive Tg B cells to the lung, as documented by recovery of anti-laminin Tg Ig in lung cell culture supernatants. Collectively, our results demonstrate that diverse disease-relevant autoreactive B cells, including anti-DNA, anti-MPO, and anti-laminin B cells, can be recruited to the lung by cSiO2 exposure, and that B cells that escape regulation can contribute to local autoantibody production in the lung. The requirement for TLR ligands to activate the autoreactive B cells further suggests that a superimposed environmental stimulus may magnify the risk of autoimmunity after cSiO2 exposure.

## Materials and methods

### Mice

Young adult autoimmune female BXSB mice were purchased from Jackson Labs and used at 1.6 months of age. Individual mice were randomly assigned to silica or vehicle instillation prior to tissue harvest 2 or 3 months later. A subset of BXSB mice (n=12) were also administered 50 μg lipopolysaccharide (LPS) (UltraPure LPS-B5, *In vivo*gen, San Diego, CA) in 500 μL PBS intraperitoneally (i.p.) 24 or 48 hours before harvest at 3 months after cSiO2 or vehicle exposure. Lung histopathology and serum and BALF autoantibody levels for the subset of BXSB mice not subjected to i.p. LPS administration were previously reported, and Materials and Methods are more fully described in that study from our lab (25).

Generation and characterization of the LamH IgMa+ autoAb Tg DNA construct and the aberrant M7 autoAb Tg line expressing the dominant anti-laminin LamH Ig heavy chain were previously described (39, 40). To restrict Tg antibody specificity, a subset of M7 autoAb Tg mice were bred to heterozygosity (Tg/KI) for the Vk8/Jk5 V8R Ig light chain (LC) knock-in (43). The M7 autoAb Tg and Vk8/Jk5 V8R Ig LC knock-in were crossed onto the non-autoimmune C57BL/6J (B6) strain >20 generations. Experimental autoAb Tg mice were bred in our colony and included adult male and female mice at approximately 2.5 months of age. Mice were housed in microisolators at maximum 4 per cage with free access to food and water in a specific pathogen-free facility with constant temperature and humidity and a 14:10-h light/dark cycle. The care and use of all experimental animals were in accordance with institutional guidelines and all studies and procedures were approved by the local Institutional Animal Care and Use Committees and conform to institutional standards and to the National Institutes of Health guide for the care and use of laboratory animals.

### Silica administration

Heat-sterilized endotoxin-free crystalline silica (Min-U-Sil-5, average particle size 1.5-2 μm) was kindly donated by Andrew Ghio, Ph.D, and administered once by oropharyngeal instillation as a suspension in sterile phosphate-buffered saline at 0.2 mg/gm (4 mg per 20 gm mouse) in a total volume of 60 μl, as described (25). This dose of silica was chosen because it results in substantial delivery of cSiO2 to the lungs with resultant lung inflammation and is consistent with the total dose used by Bates et al. to approximate half of a human lifetime exposure to respirable cSiO2 based on occupational health guidelines (23). Saline alone was administered as vehicle control.

### Tissue and organ harvest and cell preparation and culture

Mice were sacrificed at indicated times after silica or vehicle administration using CO_2_ euthanasia and organs harvested as previously described (25). Briefly, blood was collected, and serum stored at -20°C. The spleen was removed, cells dissociated, and red blood cells lysed prior to culture. After organ perfusion with saline *via* the right ventricle and trachea cannulation, lungs were lavaged with PBS and BALF collected, centrifuged, and supernatant stored at -80°C. Cells in the BALF cell pellet were enumerated using a Cellometer K2 (Nexcelom Bioscience, Laurence, MA). The right lung was removed, digested with collagenase and DNase I, cells collected through a 70 μm strainer, and red blood cells lysed prior to culture. The left lung was inflated with 10% formalin to a pressure of 20 cm H_2_0, fixed in formalin overnight, then stored in 70% ethanol prior to paraffin-embedding and sectioning at the Duke Surgical Pathology Core.

Lung and spleen cells were cultured as described (25). Briefly, one million cells/mL were cultured in an incubator (5% CO_2_ at 37°C) for 7–8 days with medium +/- TLR ligands, at which time culture supernatants were collected and stored with 0.01% sodium azide at −20°C. Medium included RPMI 1640 (Sigma, St. Louis. MO) containing 10% heat inactivated fetal bovine serum (HI-FBS) and additives as described (25). TLR ligands included 50 μg/mL LPS (TLR4 agonist, Sigma) or a combination of 2 μg/mL resiquimod (R848, TLR7 agonist, Sigma) and 1 μg/mL ODN 1668 CpG oligos (CpG, TLR9 agonist, Invivogen, San Diego, CA).

### Quantitation of lung lymphoid follicles and injury

To enumerate lymphoid collections, deparaffinized FFPE lung sections were subject to antigen retrieval, stained for mouse CD3 and B220 using antibodies with fluorescent markers, and counterstained with DAPI to aid localization of cell clusters, as described (25). (DAPI fluorescence is omitted in figure photomicrographs to facilitate visualization of CD3 and B220 staining.) Slides were scanned at the Alafi Neuroimaging Core (Washington University, St, Louis, MO) and lymphoid collections identified, outlined, and enumerated on whole lung sections using NDP Viewer software (Hamamatsu) as described (25), by an investigator blinded to experimental study group. Lung injury was scored on H&E or PAS stained tissue using a 5-point scale by a veterinarian pathologist blinded to experimental conditions, as described (25).

### Quantitation of autoantibodies and Ig concentration by ELISA

Ig concentrations, anti-DNA and anti-MPO IgM and IgG, and anti-laminin IgMa autoantibodies in BALF, culture supernatants, and serum were determined by ELISA, as described (25, 44). Autoantibody levels are reported as OD405 for binding to antigen minus binding to diluent-only using samples in duplicate. BALF and cell culture supernatants were assayed undiluted, and serum was diluted as indicated in the text and figure legends.

### Statistical analysis

Differences between groups were assessed using the Wilcoxon rank sum test, with p<0.05 considered significant. Analyses were performed using JMP software (SAS, Cary, NC). Data are reported as mean ± SD unless otherwise indicated.

## Results

### Lung inflammation, lymphoid aggregates, and autoantibodies in female BXSB mice exposed to cSiO2 with and without intraperitoneal TLR4 ligand

**Figure 1:**
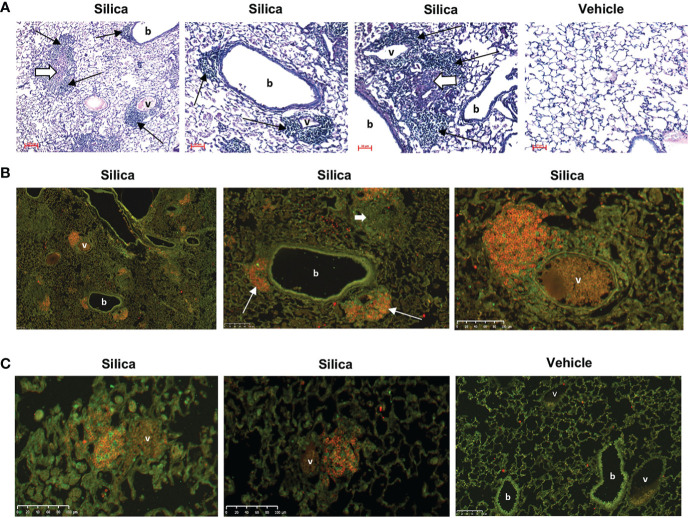
Lung inflammation and lymphocyte infiltration in female BXSB mice exposed to respiratory crystalline silica or vehicle and i.p. lipopolysaccharide (LPS). **(A)** Representative sections of lungs from mice exposed by oropharyngeal aspiration (OPA) at 3 months, and to Toll-like receptor-4 ligand LPS by i.p. injection at 2 days, prior to organ harvest. PAS, magnification indicated by micron bar. Thin black arrows indicate ectopic lymphoid aggregates; short thick white arrows indicate chronic inflammation. **(B, C)** Immunofluorescence staining of ectopic lymphoid structures in lung sections from three different mice exposed to silica, and one mouse exposed to vehicle (saline), by OPA and to LPS i.p. injection. Lung sections were stained with anti-B220 (B cells, red) and anti-CD3e (T cells, green); b = bronchiole; v = blood vessel. **(B)** Images are derived from the same silica-exposed mouse; the two right images show details within the lower power image on the left. Two lymphoid structures each encompassing a small blood vessel and adjacent to a bronchiole are indicated by thin white arrows. Diffuse lung injury and inflammation are also evident in the lung sections; the short thick white arrow indicates a granuloma.

To determine if B cells recruited to the lungs of cSiO2-exposed BXSB mice contribute to local autoantibody production, we studied tissues collected from 26 female BXSB mice exposed to cSiO2 or saline. This includes 14 female BXSB mice for which organ harvest, lung, BALF, and serum phenotype were described in our previous report (25) as well as an additional 12 female BXSB mice exposed and studied concurrently with the original cohort, but also injected i.p. with the TLR4 ligand, LPS, at 24 or 48 hours before organ harvest. The LPS injection was to test if a superimposed systemic secondary environmental stimulus mimicking bacterial infection would unmask or enhance humoral immunity associated with cSiO2 exposure in the lupus-prone BXSB mice. The subset of cSiO2-exposed mice administered i.p. LPS demonstrated extensive lung inflammation and injury and ectopic lymphoid structures (**Figure 1**), similar to histopathologic findings reported in their LPS un-injected cSiO2-exposed littermates (25). Analysis revealed little difference in measured parameters between mice that did and did not receive i.p. LPS within 48 hours prior to harvest (not shown). Based on these results, all subsequent studies to assess autoantibody production in cultured lung cells are pooled regardless of i.p. LPS administration. For these groups (cSiO2 or vehicle, n=13 per group), comparison of key disease features confirms that lung damage and inflammation are limited to the cSiO2-exposed mice, as demonstrated by lung injury scores (5.0 ± 4 vs. 0.1 ± 0.4, p<0.0001), BALF cell counts (1.29 ± 0.75 vs. 0.03 ± 0.02, in millions, p<0.0001), and number of lung lymphoid aggregates (36.1 ± 18.6 vs. 0 ± 0, p<0.0001), cSiO2 vs. vehicle (**Figures S1 A, B**).

To assess effects of cSiO2 exposure on local and systemic humoral autoimmunity in BXSB female mice, we measured autoantibodies in BALF and serum. Levels of anti-MPO IgM (OD405: 0.071 ± 0.043 vs. 0.005 ± 0.013, p<0.0001) and anti-DNA IgG (OD405: 0.588 ± 0.470, vs. 0.034 ± 0.031, p<0.0001) were significantly higher in BALF of cSiO2- compared to vehicle-exposed mice (**Figure S1C**). Levels of anti-DNA IgG were also significantly higher in serum (OD405: 0.706 ± 0.680 vs. 0.261 ± 0.182, p<0.05) of cSiO2-exposed mice (**Figure S1D**). Anti-MPO IgM autoantibodies were also detected in serum, but levels were not different between cSiO2- and vehicle-exposed mice (OD405: 0.110 ± 0.174, vs. 0.053 ± 0.054, p=0.8917), suggesting that the higher anti-MPO Ig levels observed in BALF of cSiO2-exposed mice arose from local lung autoantibody production.

### Autoantibody production by lung and spleen cells from cSiO2-exposed BXSB female mice

To assess potential locations of autoantibody production induced by cSiO2 exposure, we measured spontaneous and TLR-ligand-induced autoantibodies produced by cells isolated from lungs and spleens. Cells were harvested, washed, and plated with medium alone or with ligands to TLR4 or to both TLR7 and TLR9, using ligands previously determined to induce optimal autoantibody production from responding cell cultures (25).

**Figure 2:**
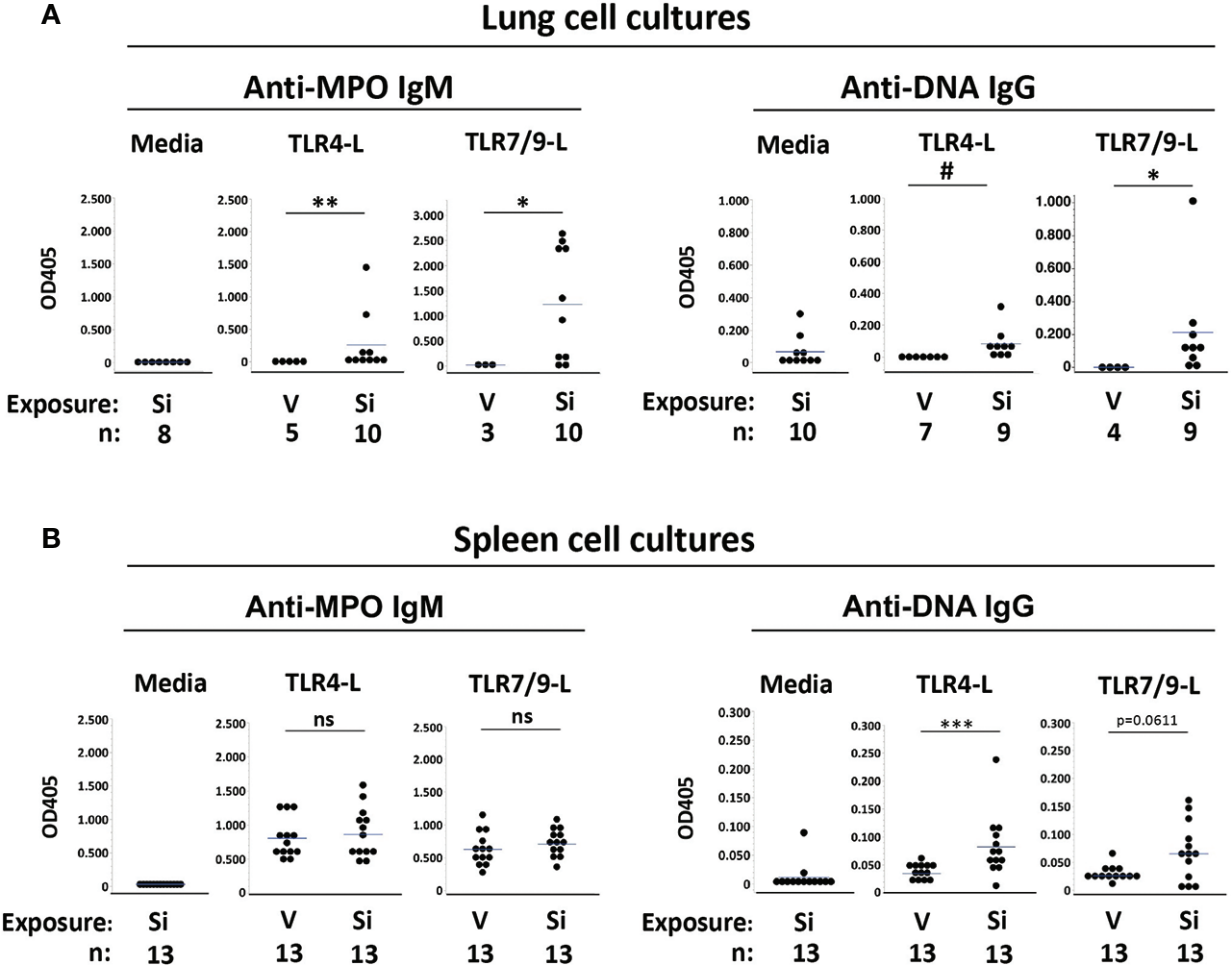
Autoantibodies produced by cultured lung and spleen cells from female BXSB mice exposed to respiratory crystalline silica or vehicle, with or without intraperitoneal lipopolysaccharide (LPS). Culture supernatants from isolated **(A)** lung cells and **(B)** splenocytes were assayed for anti-MPO IgM and anti-ssDNA IgG levels. Cells were cultured with medium alone or with either TLR4 ligand (TLR4-L) LPS or a mixture of TLR7 and TLR9 ligands (TLR7/9-L) R848 and CpG oligos, from mice of indicated exposure. Results are shown for all exposed mice, including those with or without i.p. LPS administered 1-2 days prior to organ harvest. Each circle represents an individual mouse; the bar indicates the mean for each group; *p < 0.05; **p < 0.01; ***p < 0.001; # p < 0.005; ns, not significant; for silica- vs. vehicle-exposed mice, Wilcoxon rank sum test.

Anti-MPO IgM were not detected in medium from unstimulated lung cells (**Figure 2A**). Anti-MPO IgM were also not detected in supernatants from TLR-stimulated lung cells extracted from mice exposed to vehicle (**Figure 2A**, left panels). Conversely, anti-MPO IgM were detected in most supernatants of lung cells extracted from cSiO2-exposed mice and stimulated *in vitro* with TLR4 ligand (OD405: 0.257 ± 0.471, n=10, vs. 0.0 ± 0.0, n=5, cSiO2 vs. V, p<0.01) or the TLR7/TLR9 ligand mixture (OD405: 1.232 ± 1.100, n=10, vs. 0.025 ± 0.043, n=3, p<0.05), in some instances reaching high levels (**Figure 2A**). These results of lung cell cultures indicate that anti-MPO autoreactive B cells are recruited to the lungs of cSiO2-exposed BXSB female mice and can produce autoantibody locally upon stimulation.

Low levels of anti-DNA IgG were detected in medium from unstimulated lung cells from cSiO2-exposed mice (OD405: 0.067 ± 0.095, n=10), suggesting the presence of preexisting activated anti-DNA IgG B cells or plasma cells in the BXSB cSiO2-damaged lungs (**Figure 2A**, right panels). For these cSiO2-exposed mice, anti-DNA IgG levels in supernatants from TLR4 ligand-stimulated lung cells were similar to those of unstimulated cells (OD405: 0.084 ± 0.096, n=9) (**Figure 2A**). Higher levels of anti-DNA IgG were recorded in supernatants from TLR7/TLR9 ligand-stimulated cells (OD405: 0.213 ± 0.310, n=9). In contrast, no autoantibody was detected in supernatants from TLR ligand-stimulated lung cell cultures from vehicle-exposed mice (OD405: 0.0 ± 0.0, n=7, for TLR4, and 0.001 ± 0.001, n=4, for TLR7/TLR9; p<0.005 and p<0.05, respectively, vs. cSiO2-exposed mice) (**Figure 2A**). Thus, anti-DNA IgG B cells are also recruited to cSiO2-exposed BXSB lungs.

To determine if exposure to instilled cSiO2 had systemic effects on humoral autoimmunity, we assessed autoantibody levels in spleen cell culture supernatants. Little anti-MPO or anti-DNA autoantibody was recovered from unstimulated splenocytes (**Figure 2B**, left panels). In contrast, TLR ligand stimulated abundant anti-MPO IgM from splenocytes; however, levels did not differ between the cSiO2 vs. vehicle exposure groups: OD405, 0.863 ± 0.351 vs. 0.806 ± 0.287, p=NS, for TLR4, and 0.707 ± 0.200 vs. 0.628 ± 0.257, p=NS, for TLR7/TLR9 **(** **Figure 2B**). Notably, substantial levels of anti-MPO Ab were recovered from splenocytes of all BXSB mice tested, irrespective of cSiO2 exposure, indicating the presence of numerous non-tolerized anti-MPO B cells in the BXSB strain.

Low but significantly higher levels of anti-DNA IgG were measured in TLR4 ligand-stimulated supernatants from splenocytes of cSiO2 compared to vehicle-exposed mice (OD405: 0.082 ± 0.054 vs. 0.035 ± 0.011, p<0.001), and there was a trend for higher anti-DNA IgG levels after TLR7/9-L stimulation (OD405: 0.065 ± 0.052 vs. 0.026 ± 0.014, p=0.0611) (**Figure 2B**, right panels).

### Silica exposure in B6 mice bearing an aberrantly-regulated M7 autoAb transgene (Ig Tg)

Our results showing anti-MPO and anti-DNA autoantibody production by B cells recovered from lungs of cSiO2-exposed female BXSB mice mirror those in cSiO2-exposed MRL/MpJ mice, a genetically distinct lupus-prone strain for which anti-DNA IgG, but not anti-MPO IgM, are detected in supernatants of lung cell cultures (25). Collectively the findings indicate that cSiO2 exposure recruits autoreactive B cells of diverse specificities to the lungs in the context of an autoimmune-prone background, with strain-dependent differences in autoantigen specificity reflecting genetic contributions to the final phenotype. These findings contrast with those from cSiO2-exposed non-autoimmune C57BL/6 mice, for which lung cell anti-DNA IgG production is low despite presence of abundant pulmonary lymphoid aggregates and CD19+ B cells and using *in vitro* TLR ligand stimulation (25).

To further examine the relative role of an intrinsic B cell defect versus a broader genetic autoimmune predisposition in the immune dysregulation that facilitates local autoantibody production after cSiO2 exposure, we leveraged the aberrant M7 autoantibody transgenic (Tg) mouse line. This unique mouse line was derived from the M7 founder, generated using the LamH anti-laminin IgM DNA construct. Notably, three other concurrently generated LamH Tg founder lines had a different phenotype, in which the anti-laminin Ig Tg B cells were tightly regulated by tolerance mechanisms, including deletion, anergy, and light chain editing, when bred on the C57BL/6 background (44, 45). In the non-M7 Ig Tg lines in which Tg B cell tolerance is intact, Tg autoantibodies are rarely detected in serum or in spleen cell supernatants, including in Tg mice exposed to cSiO2 (25). However, in contrast to the non-M7 lines, Tg B cells in mice in the aberrant M7 line have decreased B cell surface Tg expression and demonstrate escape from tolerance when expressed in C57BL/6 mice (39, 40). By exposing C57BL/6 M7 Tg mice to cSiO2, we can test the capacity of silica exposure to recruit dysregulated autoreactive B cells to the lung within the context of a healthy (non-lupus-prone) genetic background, as well as test the lung recruitment of B cells with a distinct auto-specificity (anti-basement membrane laminin).

In the current study, all experimental M7 Tg mice express the M7 anti-laminin autoAb Tg IgM heavy chain. This is a dominant Ig heavy chain previously shown to confer anti-laminin reactivity to a large number of B cells when paired with the diverse repertoire of endogenous Ig light chains generated by wildtype C57BL/6 LC genomic loci (40). In the current studies, mice expressing the M7 autoAb Tg with wildtype endogenous light chains are termed Tg/WW. Tg Ig are readily detected using anti-IgMa allotype reagents, because the endogenous IgM expressed in C57BL/6 mice are IgMb allotype.

**Figure 3:**
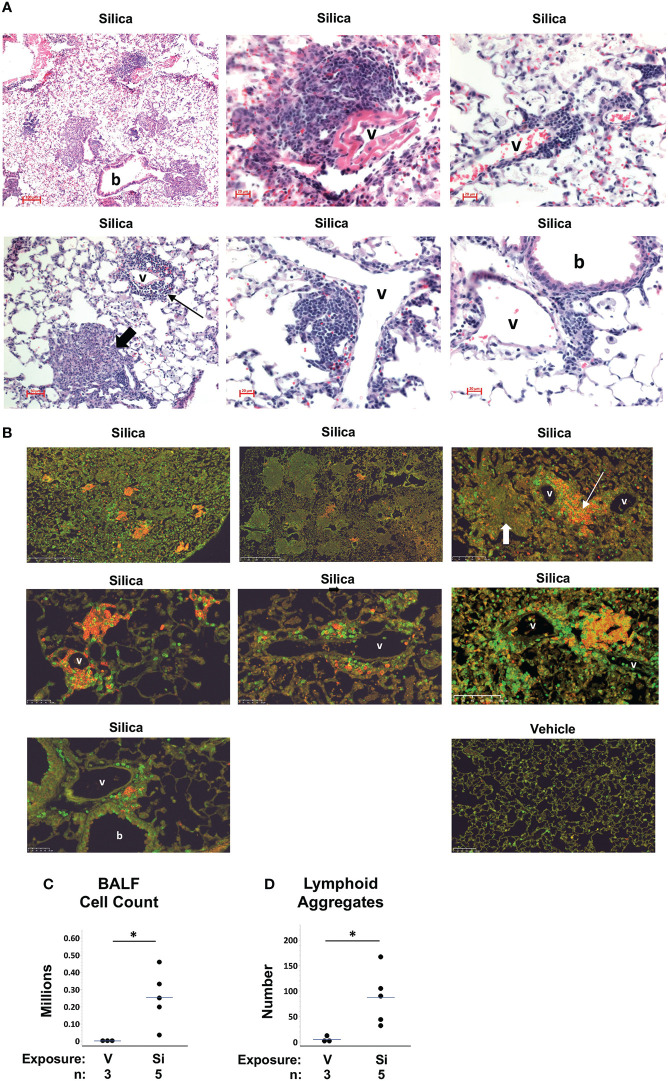
Lung inflammation and lymphoid aggregates in lungs of autoantibody M7 transgenic (Tg) Tg/WW mice exposed to respiratory crystalline silica or vehicle. Representative sections of lungs from M7 Tg/WW mice exposed to silica or saline (vehicle) by oropharyngeal aspiration at 5 to 6 weeks prior to organ harvest are stained with: **(A)** H&E; and, **(B)** Anti-B220 (B cells, red) and anti-CD3e (T cells, green); overlap of B and T cells yields yellow color in some sections. Magnification is indicated by micron bar. Lung sections from 3 different silica-exposed Tg/WW mice are shown in **(A)** and from 3 different silica-exposed mice and a vehicle-exposed mouse in **(B)**. Example lymphoid aggregates are indicated by the thin arrows and example granulomas by the short thick arrows; b = bronchiole; v = blood vessel. In **(A)** the middle image in the top row shows detail within the lower power image on its left. **(C)** Leukocyte counts in bronchoalveolar lavage fluid (BALF). **(D)** Number of lymphoid structures per whole lung section. For scatterplots each circle represents an individual mouse; the bar indicates the mean for each group. *p < 0.05; for silica- vs. vehicle-exposed mice, Wilcoxon rank sum test.

To evaluate the impact of respiratory silica exposure in M7 autoAb Tg mice, all mice were exposed to cSiO2 or vehicle by OPA concurrently and tissues were harvested 5.3 to 5.9 weeks later. This time point was chosen because we previously observed extensive lung injury by one month after cSiO2 exposure in wildtype C57BL/6 mice (25). Analysis of M7 Tg/WW mice revealed that mice that received saline (vehicle) by OPA demonstrated little lung damage (**Figure 3B**) a finding confirmed by quantitation of BALF leukocytes (**Figure 3C**) and lymphoid aggregates (**Figure 3D**). In contrast, lungs of M7 Tg/WW mice instilled with cSiO2 demonstrated extensive inflammation, granuloma formation, hemorrhage, and alveolar proteinosis as well as infiltration by lymphocytes and development of perivascular and peribronchial lymphoid aggregates (**Figures 3A–D**).

**Figure 4:**
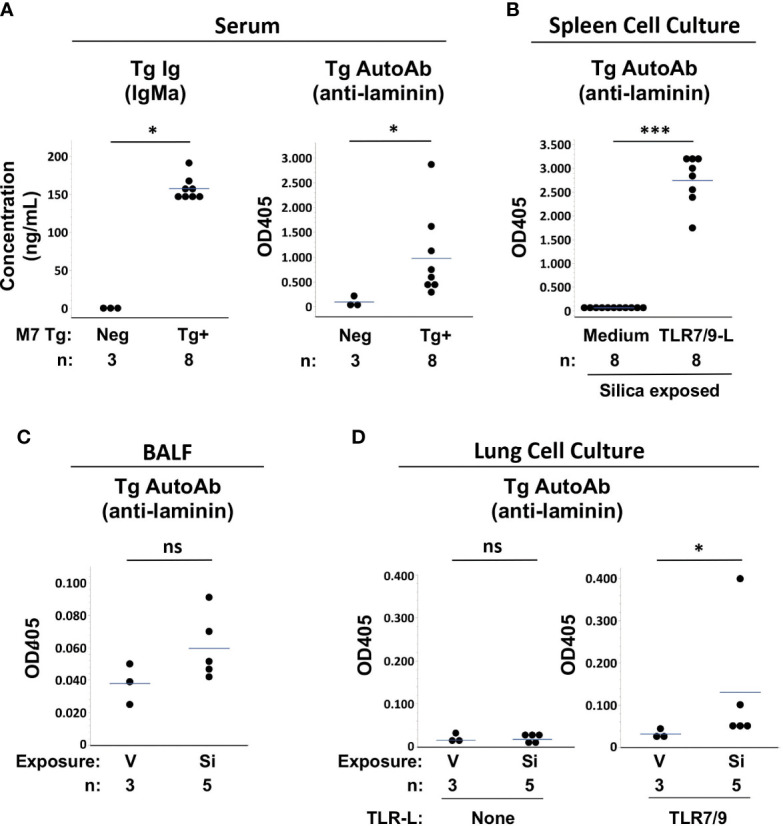
Autoantibody production in silica-exposed autoantibody transgenic (Tg) M7 Tg/WW mice. **(A)** Serum, **(B)** Spleen cell culture supernatants, **(C)** Bronchoalveolar lavage fluid (BALF), and **(D)** Lung cell culture supernatants were assayed for transgene IgMa concentration or transgene anti-laminin IgMa autoantibody levels by ELISA. In **(A)** serum was obtained at the time of organ harvest for cSiO2- and vehicle-exposed Tg/WW mice; sera from 3 non-transgenic littermates were used as negative control for Tg Ig. Autoantibodies were measured in samples in duplicate using undiluted BALF and culture supernatants and serum diluted 1/20 for anti-laminin Ig. Isolated spleen and lung cells were cultured with medium alone or with a mixture of TLR7 and TLR9 ligands (TLR7/9-L) R848 and CpG oligos. Each circle represents an individual mouse; the bar indicates the mean for each group; *p < 0.05; ***p < 0.001; ns, not significant; Wilcoxon rank sum test.

Measurement of Tg antibodies in serum confirmed that Tg/WW mice expressed a phenotype of impaired tolerance. Tg/WW mice had high levels of Tg-encoded serum IgMa and most had high levels of Tg anti-laminin IgMa autoantibodies, regardless of cSiO2 or saline exposure, compared to background levels in non-transgenic littermates, which have no Tg Ig (**Figure 4A**). The presence of abundant autoreactive Tg B cells in the Tg/WW mice was also suggested by high levels of Tg autoantibody detected in supernatants of TLR-ligand stimulated spleen cells, regardless of cSiO2 exposure (**Figure 4B**). Serum and spleen cell supernatant Tg autoantibody levels were not different between vehicle and cSiO2-exposed Tg/WW mice (p>0.05, not shown), consistent with the presence of abundant non-tolerized (dysregulated) anti-laminin autoreactive B cells in these mice.

Low levels of Tg autoAb were detected in BALF of several Si-exposed Tg/WW mice, though overall levels did not differ significantly from V-exposed mice (OD405: 0.060 ± 0.021 vs. 0.038 ± 0.013, p=0.1011) (**Figure 4C** **).** The presence in cSiO2-exposed lungs of Tg autoreactive B cells was determined by incubation of isolated lung cells with TLR7/9 ligand. Low to modest levels of Tg autoAb were detected in culture supernatants of cSiO2-exposed mice, with overall levels higher compared to cultures from V-exposed Tg/WW mice (OD405: 0.131 ± 0.152 vs. 0.032 ± 0.011, p<0.05) (**Figure 4D**). Thus, anti-laminin M7 Tg B cells are recruited to the lungs of C57BL/6 mice by cSiO2 exposure and can contribute to local autoantibody production if stimulated.

A role for intrinsic B cell dysregulation in the autoantibody production by lung B cells in the M7 Ig Tg mice is also supported by results in a mutant variant of the M7 Tg line. A subset of M7 Tg mice (termed Tg/KI) were bred to heterozygosity for a productively rearranged Vk8/Jk5 Ig light chain knock-in (KI) (43). Thus, Tg/KI mice express the M7 autoAb Tg heavy chain as well as the kappa KI light chain at one kappa locus and the wildtype unrearranged light chain allele at the second kappa locus. Combination of an Ig light chain encoded by this KI construct with the Ig heavy chain encoded by the LamH construct *in vitro* yields Ig crossreactive with laminin and DNA (44). In mice bearing both the autoAb Tg heavy chain and light chain KI, the B cell repertoire is restricted to primarily this specificity. As previously reported, in Tg+Vk8/Jk5 mouse lines derived from founders other than M7, B cell tolerance is intact and Tg autoantibodies are rarely recovered, indicating that dual Ig Tg B cells are highly regulated in this setting (44).

**Figure 5:**
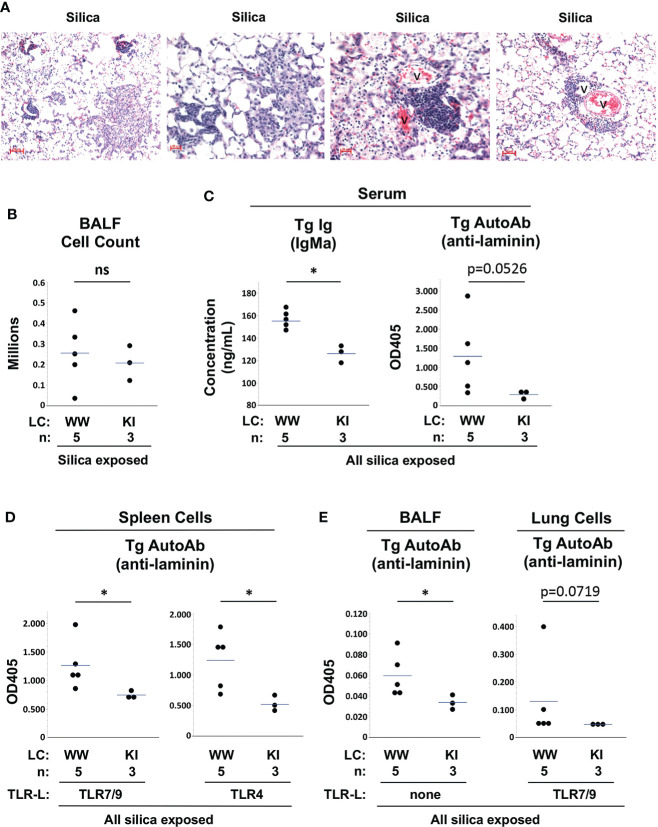
Lung inflammation and autoantibody production in silica-exposed M7 Tg/WW versus M7 Tg/KI mice. **(A)** Representative sections of lungs from 3 different M7 Tg/KI mice exposed to silica by oropharyngeal aspiration at 5 to 6 weeks prior to organ harvest; H&E; v = blood vessel. **(B)** Leukocyte count in bronchoalveolar lavage fluid (BALF). **(C–E)** Transgene IgMa concentration and anti-laminin IgMa autoantibody levels in **(C)** serum, **(D)** supernatants of spleen cell cultures, and **(E)** BALF and supernatants of lung cell cultures. Antibody levels were determined by ELISA for samples in duplicate using undiluted BALF and culture supernatants and serum diluted 1/20 for anti-laminin Ig. Isolated spleen and lung cells were cultured with a mixture of TLR7 and TLR9 ligands (TLR7/9-L) R848 and CpG oligos. All M7 mice carry the dominant LamH Ig heavy chain Tg that encodes anti-laminin Ig. Tg/WW mice carry the wildtype (W) Ig light chain gene at both kappa loci, enabling a diverse light chain repertoire; Tg/KI mice are heterozygous for a Vk8Jk5 kappa light chain knock-in (KI) gene, restricting the expressed light chain repertoire. Each circle represents an individual mouse; the bar indicates the mean for each group; *p < 0.05; Wilcoxon rank sum test.

M7 Tg/KI mice in the current studies were littermates of and studied concurrently with the Tg/WW mice described above. Similar to Tg/WW mice, M7 Tg/KI mice develop lung inflammation, lymphoid aggregates, and elevated BALF cell counts after cSiO2 exposure (**Figures 5A, B** **)**. Measurement of Tg autoantibody levels in serum and spleen cell supernatants suggest that tolerance is better maintained in M7 Tg/KI mice with a more restricted B cell repertoire. Serum Tg Ig levels are significantly lower compared to M7 Tg/WW mice (ng/mL, 126 ± 7 vs. 155 ± 9, KI vs. WW, p<0.05), and there is a trend to lower serum Tg autoAb levels (OD405: 0.293 ± 0.112 vs. 1.291 ± 1.016, KI vs. WW, p=0.0526) (**Figure 5C**). Tg autoantibody levels are also significantly lower in supernatants of TLR ligand-stimulated spleen cell cultures (OD405: 0.746 ± 0.066 vs. 1.262 ± 0.428, TLR7/9, p<0.05; and 0.515 ± 0.134 vs. 1.239 ± 0.467, TLR4, p<0.05; KI vs. WW) (**Figure 5D**). These findings are mirrored in lung autoantibody levels in cSiO2-exposed Tg/KI vs. Tg/WW mice. Tg autoantibodies are significantly lower in BALF (OD405: 0.034 ± 0.007 vs. 0.060 ± 0.021, p<0.05) and trend toward lower in supernatants of TLR ligand-stimulated lung cells (OD405: 0.048 ± 0.002 vs. 0.131 ± 0.152, p=0.0719; KI vs. WW) (**Figure 5E**). These results in Tg/KI mice in which Tg B cells appear better regulated are reminiscent of those in wildtype C57BL/6 mice. Thus production of autoantibodies from lung-recruited B cells is limited to Tg/WW mice with substantially impaired B cell tolerance.

## Discussion

In this study we used two murine models, lupus-prone BXSB mice and an autoantibody Tg line carried on the C57BL/6 background, to probe the contribution of lung-recruited B cells and genetic background to local autoantibody production after inhalation to cSiO2. Mice in both models spontaneously produce autoantibodies due in part to B cell-intrinsic impaired tolerance. We find that superimposed SiO2 exposure elicits abundant B cell-rich pulmonary lymphoid follicles in both models. Additional observations provide new insight into silica’s role in promoting autoimmunity. Using cultures of lung cells isolated from exposed mice and assayed for secreted autoantibodies, we directly demonstrate that autoreactive B cells are recruited to the cSiO2-damaged lungs. These include B cells with diverse auto-specificities implicated in silica-associated diseases, including reactivity to soluble and basement membrane proteins as well as nucleic acid. Autoantigen specificity of recruited B cells in BXSB mice is distinct from that previously reported in cSiO2-exposed MRL mice, a genetically and immunologically distinct lupus-prone strain, providing evidence that genetic background influences the phenotype of silica-exacerbated lupus. Autoantibodies are also produced by stimulated lung B cells from cSiO2-exposed autoAb M7 Tg C57BL/6 mice, indicating that a concomitant autoimmune genetic background is not essential for lung cell recruitment of dysregulated autoreactive B cells. Recruited lung B cells are induced to produce autoantibodies *in vitro* by stimulation with TLR ligands. Collectively, these findings indicate that the lung milieu created by cSiO2 exposure attracts and supports survival of dysregulated autoreactive B cells that can be readily induced to produce autoantibodies.

In both models used in these studies, autoreactive B cells escape tolerance *in vivo* by mechanisms that include intrinsic B cell defects. Defective immune regulation in BXSB mice is due to underlying polygenic autoimmune susceptibility that has been mapped to at least six non-MHC autosomal loci (46). A locus in the telomeric region of chromosome 1 overlaps with susceptibility loci from other lupus-prone strains, including the NZM2410-derived Sle1 and NZB-derived Nba2 loci that have been associated with loss of B cell tolerance in experiments using congenic mouse strains (47, 48). B cell intrinsic abnormalities identified in BXSB, as well as in genetically distinct lupus strains, include increased phosphorylation of Akt and other molecules involved in B cell receptor signaling and activation (49). An unknown mechanism, presumably a consequence of the transgene random insertion and associated with Ig allelic inclusion, is engaged in aberrant B cell tolerance in the M7 autoAb Ig Tg mouse line carried on the non-autoimmune C57BL6 background (39). Our experiments with cSiO2 exposure in BXSB and autoAb Tg mice suggest that autonomous B cell dysregulation facilitates cSiO2-enhancement of humoral autoimmunity in the lung. Preexisting dysregulation is key for autoantibody production by recruited lung B cells in the Ig Tg model. Whereas cSiO2 exposure recruits autoAb Tg B cells to lungs of M7 Tg mice, as shown here, as well as to lungs of mice bearing the anti-laminin autoAb Tg but in which the Tg B cells are tightly regulated, as we previously reported (25), autoantibody production by stimulated cultured lung cells is observed only with the M7 Tg line with the aberrant B cell tolerance phenotype.

While preexisting B cell dysregulation facilitates local autoantibody production after cSiO2 exposure, it is likely that neither preexisting dysregulation, auto-specificity, nor B cell activation are necessary for the lung recruitment of these autoreactive B cells. Denton et al. demonstrated using an anti-hen egg lysozyme (HEL) Ig Tg model that naive antigen-irrelevant anti-HEL B cells are readily recruited to influenza-induced lung lymphoid follicles in non-autoimmune mice (50). Thus, lung recruitment occurs independent of B cell receptor specificity or signaling. Our demonstration that B cells of diverse auto-specificities are recruited to BXSB and M7 Tg mouse lungs is consistent with this observation. B cell accumulation in the lung also occurs in bone marrow-reconstituted Rag2-/-IL2rg-/- mice lacking lymph nodes, further indicating that lymph node trafficking and activation are dispensable for lung lymphoid aggregate formation (50). In the influenza virus-infected lung, B cell recruitment relies on type 1 interferon-driven induction of the CXCL13 chemokine (50). It remains to be determined if similar cytokine and chemokine circuits drive B cell recruitment in lungs exposed to cSiO2 or within the autoimmune milieu of lupus-susceptible mice or individuals. It is nonetheless notable that a sustained interferon signature and CXCL13 expression have been identified in the lung transcriptome of cSiO2-exposed lupus-prone NZBWF1 mice (36).

Our experiments with cSiO2 exposure and local autoantibody production in BXSB and M7 autoAb Tg mice raise the possibility that humoral autoimmunity after cSiO2 exposure may also be facilitated in previously healthy (non-autoimmune) individuals who develop a superimposed transient defect in tolerance. In this regard, acquired defects in negative selection of autoreactive cells associated with autoantibody production have been described after exposure to a variety of environmental agents. Exposure to one of over 80 drugs of dissimilar chemical structure can precipitate reversible SLE-like autoimmunity (51). Multiple mechanisms for drug-induced loss of tolerance have been invoked. Mazari et al. demonstrated that preculture of bone marrow cells with hydralazine, a common anti-hypertension drug known to cause human lupus, induces high titers of anti-nucleosome autoantibodies in syngeneic recipients in a humanized Ig mouse model (52). Hydralazine was shown to impair B cell receptor editing, a major mechanism of B cell tolerance that is also impaired in some patients with SLE and RA (52). Hydralazine and procainamide, an antiarrhythmic medication, inhibit human CD4+ T cell DNA methylation and alter expression of immune regulatory genes (53). Adoptive transfer of procainamide-treated mouse CD4+ T cells elicits lupus-like autoantibodies and glomerulonephritis *in vivo* (54). Additional postulated mechanisms underlying drug-induced loss of tolerance include molecular mimicry, presentation of cryptic self-antigens, and disruption of thymocyte selection resulting in specific induction of chromatin-reactive T cells and IgG autoantibodies (55). A variety of viral infections are also associated with autoantibody production, indicating transient immune dysregulation and an acquired window of autoimmune susceptibility. Proposed mechanisms include virus-induced lymphopenia that depletes regulatory lymphocytes, epitope spreading, superantigen activation of T cells, activation of bystander T helper cells, in part as a manifestation of the cytokine storm, and expansion of autoimmune-prone extrafollicular B cells (56, 57). It is thus plausible that a transient acquired tolerance defect could magnify the risk of autoimmunity from cSiO2 exposure in non-autoimmune individuals.

The requirement for TLR ligands to stimulate autoantibody production by the cultured lung cells further suggests that cSiO2-promoted autoimmunity can be unmasked by a superimposed immune stimulus. We stimulated cell cultures with TLR ligands known to be potent mouse B cell activators, including LPS, a potent polyclonal B cell activator (58), and a combination of ligands to TLR7 and TLR9, based on previous evidence of synergy between these two endosomal TLRs in activating B cells in the mouse cell culture assay (25). TLR7 and TLR9 bind ssRNA and dsDNA, respectively, and have been demonstrated to support enhanced activation of B cells specific for these nuclear antigens, *via* antigen-IgG immune complexes that signal both the B cell receptor *via* antigen and the endosomal TLR after Fc-receptor-mediated endocytosis of the immune complex (59, 60). Notably, elimination of endosomal TLR signaling in multiple lupus-prone mouse strains, including BXSB, not only abolishes anti-nuclear IgG production and disease but also partially and significantly blocks production of antibodies to nonnuclear lupus-related autoantigens, including MPO, cardiolipin, and beta2-glycoprotein1 in MRL/lpr mice and red blood cells in NZB mice (61, 62). Results from mixed bone marrow chimeric mice further indicate that the major effect of endosomal TLR signaling on autoantibody production *in vivo* is B cell-intrinsic (62). Thus, a mouse or individual with a cSiO2-damaged lung containing lymphoid aggregates populated by autoreactive B cells may be particularly susceptible to local B cell activation and autoantibody production if subsequently exposed to an appropriate cellular pattern recognition receptor ligand. Common sources of exogenous TLR ligand include microbial infection or inhalation of airborne environmental allergens that either directly activate TLR or are contaminated with TLR ligands such as LPS or peptidoglycan (63). The cSiO2-damaged lung tissue itself is a potential source of endogenous TLR ligand. However, the absence of spontaneous autoAb production in supernatants from cells cultured with medium alone suggests that endogenous TLR ligands have limited influence in the experimental conditions used here. Alternatively, local autoreactive cell activation may be held in check *in vivo*, and autoantibody production averted, by immunosuppressive influences within the cSiO2-modulated lung microenvironment. Consistent with this possibility, a regulatory T cell mRNA signature, CD4+Foxp3+ regulatory T cells, and immunomodulatory mediators IL-10 and TGFbeta are increased in cSiO2-damaged mouse lungs (36, 64). In this case, a strong immune stimulus that overcomes local immune regulation would be needed to trigger autoreactive cell activation (65).

Collectively, our findings and those of other investigators suggest that a multi-step model is involved in cSiO2-induced autoimmunity. It is clear that cSiO2 exposure creates lung inflammation that recruits lymphocytes and promotes formation of abundant pulmonary tertiary lymphoid follicles in diverse mouse strains, including non-autoimmune mice. Dysregulated autoreactive B cells, as well as regulated autoreactive and non-autoreactive B cells, are readily recruited to lung lymphoid aggregates. A superimposed stimulus, such as ligand to TLR introduced by inhalation, infection, or *via* the circulation, can promote B cell activation locally either directly by engaging B cell TLR or indirectly by activating innate immune cells and modulating the local immune microenvironment. Dysregulated B cells with intrinsic genetically-determined or acquired transient defects in immunological tolerance may be particularly susceptible to activation in the setting of an immunologically-relevant environmental co-exposure. Such a scenario is supported by the observations of Gonazalez-Quintial and colleagues (66). Although not focused specifically on local lung autoimmune interactions, these investigators observed elevation of circulating anti-nuclear IgG levels three months after cSiO2 instillation in C57BL/6 mice with underlying persistent lymphocytic choriomenigitis virus infection but not in virus-naive C57BL/6 mice (66).

It is interesting that injection of LPS i.p. 24 to 48 hours prior to tissue harvest had little impact on parameters measured in our studies. The rationale for this approach was to mimic a common environmental co-exposure (bacterial infection) associated with potent polyclonal B cell activation in mice (58, 67). It is possible that the *in vivo* response to LPS in injected mice was dampened by preconditioning or other effects related to the systemic inflammatory response associated with cSiO2 lung exposure (68). In this regard, elevated circulating levels of several immune mediators that modulate B cell function, including BAFF, osteopontin, and TNFalpha, have been documented in cSiO2-exposed mice (22, 23). Alternatively, our study time frame may have been too short to detect an increase in autoantibodies, although it is reported that NFkB nuclear translocation and TLR4 signaling in peripheral tissue cells peak within an hour after *in vivo* LPS injection in mice (69). It is possible that a longer interval or *in vivo* exposure to a higher dose of LPS or alternative TLR agonist(s) such as a TLR 7 or TLR 9 ligands will induce a different response in the setting of cSiO2-induced lung injury.

Our findings also raise additional questions for future study about the interaction of the humoral immune system within the cSiO2-induced lung microenvironment. The degree to which lung-recruited autoreactive B cells become activated locally or are held in check by local immunosuppressive factors requires further study. The results may explain our finding of autoantibodies in BALF but not in unstimulated cultures of lung cells from cSiO2-exposed mice. It is also not clear if the cSiO2-induced local microenvironment or systemic factors directly promote loss of tolerance, or simply amplify autoantibody production by cells that have already escaped tolerance. The stimulants (autoantigen, TLR ligands, or other factors), T cells and other immune cells, and immune microenvironments (including follicular vs. extrafollicular sites) that trigger local autoreactive B cell activation are not yet identified. The relative contribution of lung versus systemic B cells to generation of circulating autoantibodies is unknown, as is the impact of lung-derived soluble and cellular mediators on autoimmune responses in distant lymphoid organs. In this regard, different mechanisms may be operative in different genetic backgrounds. In contrast to findings in female C57BL/6, BXSB, and MRL/MpJ mice, we observed elevated serum anti-DNA IgG with no difference in BALF anti-DNA Ig levels in cSiO2-exposed lupus-prone NZB mice, suggesting that the effect on humoral autoimmunity was primarily systemic in NZB (25). Finally, the role of B cell-derived cytokines (70) on pathogenesis in cSiO2-induced autoimmunity deserves further study.

In conclusion, dysregulated autoreactive B cells of diverse disease-relevant specificities are recruited to cSiO2-exposed lungs and can contribute to local autoantibody production. A superimposed immune stimulus, such as exposure to an exogenous TLR ligand, may be necessary to unmask cSiO2-promoted autoimmunity. Elucidation of pathogenic mechanisms will provide insights into human autoimmunity and help identify novel therapeutic targets for autoimmune disease.

## Data availability statement

The original contributions presented in the study are included in the article/**Supplementary Material**. Further inquiries can be directed to the corresponding author.

## Ethics statement

The animal study was reviewed and approved by Duke University Institutional Animal Care and Use Committee and Durham VAMC IACUC.

## Author contributions

LF, RT, and MF contributed to conception and design of the study; LF, AK, and MF performed experiments; LF and MF performed statistical analyses; and LF, RT, and MF wrote the manuscript. All authors contributed to the article and approved the submitted version.

## Funding

Research reported in this publication was supported by the National Institute of Environmental Health Sciences (NIEHS) under award numbers R01ES027873 (MF) and Duke University Undergraduate Research grants (AK).

## Acknowledgments

As a Duke Cancer Institute member, MF acknowledges support from the Duke Cancer Institute as part of the P30 Cancer Center Support Grant (Grant ID: P30 CA014236). We thank Dr. Yohannes Asfaw, Division of Laboratory Animal Resources, Duke University, for analyzing mouse lung pathology. We thank Andrew Ghio of the National Health and Environmental Effects Research Laboratory, US Environmental Protection Agency, Chapel Hill, NC, for the gift of silica. We thank the Flow Cytometry Shared Resources, DNA Analysis, and Light Microscopy Facility of the Duke Cancer Institute, the Duke BioRepository & Precision Pathology Center Shared Resource, the Duke Substrate Services Core & Research Support resource, the Duke and VAMC Animal Care Facilities staff, and the Alafi Neuroimaging Core supported by the Hope Center for Neurological Disorders (Washington University in St, Louis) and an NIH Shared Instrumentation Grant (S10 RR027552).

## Conflict of interest

The authors declare that the research was conducted in the absence of any commercial or financial relationships that could be construed as a potential conflict of interest.

## Publisher’s note

All claims expressed in this article are solely those of the authors and do not necessarily represent those of their affiliated organizations, or those of the publisher, the editors and the reviewers. Any product that may be evaluated in this article, or claim that may be made by its manufacturer, is not guaranteed or endorsed by the publisher.

## Author disclaimer

The content is solely the responsibility of the authors and does not necessarily represent the official views of the National Institutes of Health.

## Supplementary material

Supplementary Figure 1 Lung inflammation, lymphocyte infiltration, and autoantibodies in bronchoalveolar lavage fluid (BALF) and serum of female BXSB mice exposed to respiratory crystalline silica or vehicle (saline), with or without intraperitoneal (i.p.) TLR4 ligand. Mice were exposed by oropharyngeal aspiration (OPA) at 2 or 3 months before tissue harvest, with or without i.p. injection of TLR4 ligand lipopolysaccharide (LPS) at 2 days prior to harvest. **(A)** Leukocyte count in BALF. **(B)** Number of ectopic lymphoid aggregates per whole lung section, quantified on FFPE lung sections stained with fluorescently-labeled anti-B220 (B cells) and anti-CD3 (T cells) antibodies. **(C, D)** Autoantibodies measured in samples in duplicate using **(C)** undiluted BALF and **(D)** serum diluted 1/100 for anti-DNA Ig and 1/20 for anti-myeloperoxidase (MPO) Ig. Each circle represents an individual mouse; the bar indicates the mean for each group; *p < 0.05; ***p < 0.0001; ns, not significant, for silica- *vs*. vehicle-exposed mice, Wilcoxon rank sum test.

## Abbreviations

ANCA, anti-neutrophil cytoplasmic autoantibodies; AAV, ANCA associated vasculitis; BALF, bronchoalveolar lavage fluid; cSiO2, crystalline silica; MPO, myeloperoxidase; NZB, New Zealand Black; RA, rheumatoid arthritis; SLE, systemic lupus erythematosus; SS, systemic sclerosis; TLR, Toll-like receptor.

## References

[1] Dinse GE Parks CG Weinberg CR Co CA Wilkerson J Zeldin DC. Increasing prevalence of antinuclear antibodies in the United States. Arthritis Rheumatol (2020) 72(6):1026–35. doi: 10.1002/art.41214 PMC7255943 32266792

[2] Farh KK Marson A Zhu J Kleinewietfeld M Housley WJ Beik S. Genetic and epigenetic fine mapping of causal autoimmune disease variants. Nature (2015) 518(7539):337–43. doi: 10.1038/nature13835 PMC4336207 25363779

[3] Parks CG Conrad K Cooper GS. Occupational exposure to crystalline silica and autoimmune disease. Environ Health Perspect (1999) 107 Suppl 5:793–802. doi: 10.1289/ehp.99107s5793 10970168 PMC1566238

[4] Miller FW Alfredsson L Costenbader KH Kamen DL Nelson LM Norris JM. Epidemiology of environmental exposures and human autoimmune diseases: Findings from a National Institute of Environmental Health Sciences expert panel workshop. J Autoimmun (2012) 39(4):259–71. doi: 10.1016/j.jaut.2012.05.002 PMC3496812 22739348

[5] Lewis EJ Busch GJ Schur PH. Gamma G globulin subgroup composition of the glomerular deposits in human renal diseases. J Clin Invest (1970) 49(6):1103–13. doi: 10.1172/JCI106326 PMC322578 4987169

[6] Termaat RM Assmann KJ Dijkman HB van Gompel F Smeenk RJ Berden JH. Anti-DNA antibodies can bind to the glomerulus *via* two distinct mechanisms. Kidney Int (1992) 42(6):1363–71. doi: 10.1038/ki.1992.428 1474767

[7] Giannouli S Voulgarelis M Ziakas PD Tzioufas AG. Anaemia in systemic lupus erythematosus: From pathophysiology to clinical assessment. Ann Rheum Dis (2006) 65(2):144–8. doi: 10.1136/ard.2005.041673 PMC1798007 16079164

[8] Hochberg MC. Updating the American College of Rheumatology revised criteria for the classification of systemic lupus erythematosus. Arthritis Rheumatol (1997) 40(9):1725. doi: 10.1002/art.1780400928 9324032

[9] Petri M Orbai AM Alarcon GS Gordon C Merrill JT Fortin PR. Derivation and validation of the systemic lupus international collaborating clinics classification criteria for systemic lupus erythematosus. Arthritis Rheumatol (2012) 64(8):2677–86. doi: 10.1002/art.34473 PMC3409311 22553077

[10] Li QZ Xie C Wu T Mackay M Aranow C Putterman C. Identification of autoantibody clusters that best predict lupus disease activity using glomerular proteome arrays. J Clin Invest (2005) 115(12):3428–39. doi: 10.1172/JCI23587 PMC1297234 16322790

[11] de Brito Rocha S Baldo DC Andrade LEC. Clinical and pathophysiologic relevance of autoantibodies in rheumatoid arthritis. Adv Rheumatol (2019) 59(1):2. doi: 10.1186/s42358-018-0042-8 30657101

[12] Yang C Tang S Zhu D Ding Y Qiao J. Classical disease-specific autoantibodies in systemic sclerosis: Clinical features, gene susceptibility, and disease stratification. Front Med (Lausanne) (2020) 7:587773. doi: 10.3389/fmed.2020.587773 33330547 PMC7710911

[13] Jennette JC Nachman PH. ANCA glomerulonephritis and vasculitis. Clin J Am Soc Nephrol (2017) 12(10):1680–91. doi: 10.2215/CJN.02500317 PMC5628710 28842398

[14] Navarra SV Guzman RM Gallacher AE Hall S Levy RA Jimenez RE. Efficacy and safety of belimumab in patients with active systemic lupus erythematosus: A randomised, placebo-controlled, phase 3 trial. Lancet (2011) 377(9767):721–31. doi: 10.1016/S0140-6736(10)61354-2 21296403

[15] Furie R Petri M Zamani O Cervera R Wallace DJ Tegzova D. A phase III, randomized, placebo-controlled study of belimumab, a monoclonal antibody that inhibits b lymphocyte stimulator, in patients with systemic lupus erythematosus. Arthritis Rheumatol (2011) 63(12):3918–30. doi: 10.1002/art.30613 PMC5007058 22127708

[16] Klimatcheva E Pandina T Reilly C Torno S Bussler H Scrivens M. CXCL13 antibody for the treatment of autoimmune disorders. BMC Immunol (2015) 16:6. doi: 10.1186/s12865-015-0068-1 25879435 PMC4329654

[17] Lee DSW Rojas OL Gommerman JL. B cell depletion therapies in autoimmune disease: advances and mechanistic insights. Nat Rev Drug Discov (2021) 20(3):179–99. doi: 10.1038/s41573-020-00092-2 PMC7737718 33324003

[18] Neys SFH Hendriks RW Corneth OBJ. Targeting bruton's tyrosine kinase in inflammatory and autoimmune pathologies. Front Cell Dev Biol (2021) 9:668131. doi: 10.3389/fcell.2021.668131 34150760 PMC8213343

[19] Brown JM Archer AJ Pfau JC Holian A. Silica accelerated systemic autoimmune disease in lupus-prone New Zealand mixed mice. Clin Exp Immunol (2003) 131(3):415–21. doi: 10.1046/j.1365-2249.2003.02094.x PMC1808650 12605693

[20] Brown JM Schwanke CM Pershouse MA Pfau JC Holian A. Effects of rottlerin on silica-exacerbated systemic autoimmune disease in New Zealand mixed mice. Am J Physiol Lung Cell Mol Physiol (2005) 289(6):L990–8. doi: 10.1152/ajplung.00078.2005 16040631

[21] Chen Y Li C Weng D Song L Tang W Dai W. Neutralization of interleukin-17A delays progression of silica-induced lung inflammation and fibrosis in C57BL/6 mice. Toxicol Appl Pharmacol (2014) 275(1):62–72. doi: 10.1016/j.taap.2013.11.012 24291675

[22] Bates MA Brandenberger C Langohr I Kumagai K Harkema JR Holian A. Silica triggers inflammation and ectopic lymphoid neogenesis in the lungs in parallel with accelerated onset of systemic autoimmunity and glomerulonephritis in the lupus-prone NZBWF1 mouse. PloS One (2015) 10(5):e0125481. doi: 10.1371/journal.pone.0125481 25978333 PMC4433215

[23] Bates MA Brandenberger C Langohr II Kumagai K Lock AL Harkema JR. Silica-triggered autoimmunity in lupus-prone mice blocked by docosahexaenoic acid consumption. PloS One (2016) 11(8):e0160622. doi: 10.1371/journal.pone.0160622 27513935 PMC4981380

[24] Bates MA Akbari P Gilley KN Wagner JG Li N Kopec AK. Dietary docosahexaenoic acid prevents silica-induced development of pulmonary ectopic germinal centers and glomerulonephritis in the lupus-prone NZBWF1 mouse. Front Immunol (2018) 9:2002. doi: 10.3389/fimmu.2018.02002 30258439 PMC6143671

[25] Foster MH Ord JR Zhao EJ Birukova A Fee L Korte FM. Silica exposure differentially modulates autoimmunity in lupus strains and autoantibody transgenic mice. Front Immunol (2019) 10:2336. doi: 10.3389/fimmu.2019.02336 31632407 PMC6781616

[26] Gilley KN Wierenga KA Chauhuan PS Wagner JG Lewandowski RP Ross EA. Influence of total western diet on docosahexaenoic acid suppression of silica-triggered lupus flaring in NZBWF1 mice. PloS One (2020) 15(5):e0233183. doi: 10.1371/journal.pone.0233183 32413078 PMC7228097

[27] Rajasinghe LD Li QZ Zhu C Yan M Chauhan PS Wierenga KA. Omega-3 fatty acid intake suppresses induction of diverse autoantibody repertoire by crystalline silica in lupus-prone mice. Autoimmunity (2020) 53(7):415–33. doi: 10.1080/08916934.2020.1801651 PMC8020726 32903098

[28] Pestka JJ Akbari P Wierenga KA Bates MA Gilley KN Wagner JG. Omega-3 polyunsaturated fatty acid intervention against established autoimmunity in a murine model of toxicant-triggered lupus. Front Immunol (2021) 12:653464. doi: 10.3389/fimmu.2021.653464 33897700 PMC8058219

[29] Mayeux JM Escalante GM Christy JM Pawar RD Kono DH Pollard KM. Silicosis and silica-induced autoimmunity in the diversity outbred mouse. Front Immunol (2018) 9:874. doi: 10.3389/fimmu.2018.00874 29755467 PMC5932595

[30] Pollard KM. Silica, silicosis, and autoimmunity. Front Immunol (2016) 7:97. doi: 10.3389/fimmu.2016.00097 27014276 PMC4786551

[31] Davis GS Pfeiffer LM Hemenway DR. Persistent overexpression of interleukin-1beta and tumor necrosis factor-alpha in murine silicosis. J Environ Pathol Toxicol Oncol (1998) 17(2):99–114. 9546746

[32] Davis GS Holmes CE Pfeiffer LM Hemenway DR. Lymphocytes, lymphokines, and silicosis. J Environ Pathol Toxicol Oncol (2001) 20 Suppl 1:53–65. doi: 10.1615/JEnvironPatholToxicolOncol.v20.iSuppl.1.50 11570674

[33] Brown JM Pfau JC Holian A. Immunoglobulin and lymphocyte responses following silica exposure in New Zealand mixed mice. Inhal Toxicol (2004) 16(3):133–9. doi: 10.1080/08958370490270936 15204774

[34] Iyer R Hamilton RF Li L Holian A. Silica-induced apoptosis mediated *via* scavenger receptor in human alveolar macrophages. Toxicol Appl Pharmacol (1996) 141(1):84–92. doi: 10.1016/S0041-008X(96)80012-3 8917679

[35] Pfau JC Brown JM Holian A. Silica-exposed mice generate autoantibodies to apoptotic cells. Toxicology (2004) 195(2-3):167–76. doi: 10.1016/j.tox.2003.09.011 14751672

[36] Bates MA Benninghoff AD Gilley KN Holian A Harkema JR Pestka JJ. Mapping of dynamic transcriptome changes associated with silica-triggered autoimmune pathogenesis in the lupus-prone NZBWF1 mouse. Front Immunol (2019) 10:632. doi: 10.3389/fimmu.2019.00632 30984195 PMC6450439

[37] Chauhan PS Wagner JG Benning hoff AD Lewandowski RP Favor OK Wierenga KA. Rapid induction of pulmonary inflammation, autoimmune gene expression, and ectopic lymphoid neogenesis following acute silica exposure in lupus-prone mice. Front Immunol (2021) 12:635138. doi: 10.3389/fimmu.2021.635138 33732257 PMC7959771

[38] Kimura J Ichii O Nakamura T Horino T Otsuka S Kon Y. BXSB-type genome causes murine autoimmune glomerulonephritis: Pathological correlation between telomeric region of chromosome 1 and yaa. Genes Immun (2014) 15(3):182–9. doi: 10.1038/gene.2014.4 24477164

[39] Foster MH Fitzsimons MM. Lupus-like nephrotropic autoantibodies in non-autoimmune mice harboring an anti-basement membrane/anti-DNA IG heavy chain transgene. Mol Immunol (1998) 35(2):83–94. doi: 10.1016/S0161-5890(98)00018-2 9683254

[40] Fitzsimons MM Chen H Foster MH. Diverse endogenous light chains contribute to basement membrane reactivity in nonautoimmune mice transgenic for an anti-laminin IG heavy chain. Immunogenetics (2000) 51(1):20–9. doi: 10.1007/s002510050004 10663558

[41] Amital H Heilweil M Ulmansky R Szafer F Bar-Tana R Morel L. Treatment with a laminin-derived peptide suppresses lupus nephritis. J Immunol (2005) 175(8):5516–23. doi: 10.4049/jimmunol.175.8.5516 16210660

[42] Amital H Heilweil-Harel M Ulmansky R Harlev M Toubi E Hershko A. Antibodies against the VRT101 laminin epitope correlate with human SLE disease activity and can be removed by extracorporeal immunoadsorption. Rheumatol (Oxford) (2007) 46(9):1433–7. doi: 10.1093/rheumatology/kem181 17686790

[43] Prak EL Trounstine M Huszar D Weigert M. Light chain editing in kappa-deficient animals: A potential mechanism of B cell tolerance. J Exp Med (1994) 180(5):1805–15. doi: 10.1084/jem.180.5.1805 PMC2191736 7964462

[44] Brady GF Congdon KL Clark AG Sackey FN Rudolph EH Radic MZ. Kappa editing rescues autoreactive B cells destined for deletion in mice transgenic for a dual specific anti-laminin IG. J Immunol (2004) 172(9):5313–21. doi: 10.4049/jimmunol.172.9.5313 15100270

[45] Rudolph EH Congdon KL Sackey FN Fitzsimons MM Foster MH. Humoral autoimmunity to basement membrane antigens is regulated in C57BL/6 and MRL/MpJ mice transgenic for anti-laminin IG receptors. J Immunol (2002) 168(11):5943–53. doi: 10.4049/jimmunol.168.11.5943 12023401

[46] Haywood ME Hogarth MB Slingsby JH Rose SJ Allen PJ Thompson EM. Identification of intervals on chromosomes 1, 3, and 13 linked to the development of lupus in BXSB mice. Arthritis Rheumatol (2000) 43(2):349–55. doi: 10.1002/1529-0131(200002)43:2<349::AID-ANR14>3.0.CO;2-M 10693874

[47] Sobel ES Mohan C Morel L Schiffenbauer J Wakeland EK. Genetic dissection of SLE pathogenesis: adoptive transfer of Sle1 mediates the loss of tolerance by bone marrow-derived b cells. J Immunol (1999) 162(4):2415–21. 9973523

[48] Chang NH Manion KP Loh C Pau E Baglaenko Y Wither JE. Multiple tolerance defects contribute to the breach of B cell tolerance in New Zealand black chromosome 1 congenic mice. PloS One (2017) 12(6):e0179506. doi: 10.1371/journal.pone.0179506 28628673 PMC5476272

[49] Wu T Qin X Kurepa Z Kumar KR Liu K Kanta H. Shared signaling networks active in B cells isolated from genetically distinct mouse models of lupus. J Clin Invest (2007) 117(8):2186–96. doi: 10.1172/JCI30398 PMC1913486 17641780

[50] Denton AE Innocentin S Carr EJ Bradford BM Lafouresse F Mabbott NA. Type I interferon induces CXCL13 to support ectopic germinal center formation. J Exp Med (2019) 216(3):621–37. doi: 10.1084/jem.20181216 PMC6400543 30723095

[51] Vasoo S. Drug-induced lupus: An update. Lupus. (2006) 15(11):757–61. doi: 10.1177/0961203306070000 17153847

[52] Mazari L Ouarzane M Zouali M. Subversion of B lymphocyte tolerance by hydralazine, a potential mechanism for drug-induced lupus. Proc Natl Acad Sci USA (2007) 104(15):6317–22. doi: 10.1073/pnas.0610434104 PMC1851062 17404230

[53] Cornacchia E Golbus J Maybaum J Strahler J Hanash S Richardson B. Hydralazine and procainamide inhibit T cell DNA methylation and induce autoreactivity. J Immunol (1988) 140(7):2197–200. 3258330

[54] Quddus J Johnson KJ Gavalchin J Amento EP Chrisp CE Yung RL. Treating activated CD4+ T cells with either of two distinct DNA methyltransferase inhibitors, 5-azacytidine or procainamide, is sufficient to cause a lupus-like disease in syngeneic mice. J Clin Invest (1993) 92(1):38–53. doi: 10.1172/JCI116576 7686923 PMC293525

[55] Kretz-Rommel A Duncan SR Rubin RL. Autoimmunity caused by disruption of central T cell tolerance. A murine model of drug-induced lupus. J Clin Invest (1997) 99(8):1888–96. doi: 10.1172/JCI119356 PMC508013 9109433

[56] Smatti MK Cyprian FS Nasrallah GK Al Thani AA Almishal RO Yassine HM. Viruses and autoimmunity: A review on the potential interaction and molecular mechanisms. Viruses (2019) 11(8):762–79. doi: 10.3390/v11080762 PMC6723519 31430946

[57] Knight JS Caricchio R Casanova JL Combes AJ Diamond B Fox SE. The intersection of COVID-19 and autoimmunity. J Clin Invest (2021) 131(24):e154886. doi: 10.1172/JCI154886 34710063 PMC8670833

[58] Venkataraman C Shankar G Sen G Bondada S. Bacterial lipopolysaccharide induced B cell activation is mediated *via* a phosphatidylinositol 3-kinase dependent signaling pathway. Immunol Lett (1999) 69(2):233–8. doi: 10.1016/S0165-2478(99)00068-1 10482357

[59] Leadbetter EA Rifkin IR Hohlbaum AM Beaudette BC Shlomchik MJ Marshak-Rothstein A. Chromatin-IgG complexes activate B cells by dual engagement of IgM and toll-like receptors. Nature (2002) 416(6881):603–7. doi: 10.1038/416603a 11948342

[60] Marshak-Rothstein A Rifkin IR. Immunologically active autoantigens: the role of toll-like receptors in the development of chronic inflammatory disease. Annu Rev Immunol (2007) 25:419–41. doi: 10.1146/annurev.immunol.22.012703.104514 17378763

[61] Kono DH Haraldsson MK Lawson BR Pollard KM Koh YT Du X. Endosomal TLR signaling is required for anti-nucleic acid and rheumatoid factor autoantibodies in lupus. Proc Natl Acad Sci USA (2009) 106(29):12061–6. doi: 10.1073/pnas.0905441106 PMC2715524 19574451

[62] Koh YT Scatizzi JC Gahan JD Lawson BR Baccala R Pollard KM. Role of nucleic acid-sensing TLRs in diverse autoantibody specificities and anti-nuclear antibody-producing B cells. J Immunol (2013) 190(10):4982–90. doi: 10.4049/jimmunol.1202986 PMC3729324 23589617

[63] Zakeri A Russo M. Dual role of toll-like receptors in human and experimental asthma models. Front Immunol (2018) 9:1027. doi: 10.3389/fimmu.2018.01027 29867994 PMC5963123

[64] Liu F Liu J Weng D Chen Y Song L He Q. CD4+CD25+Foxp3+ regulatory T cells depletion may attenuate the development of silica-induced lung fibrosis in mice. PloS One (2010) 5(11):e15404. doi: 10.1371/journal.pone.0015404 21072213 PMC2972313

[65] Rosenspire AJ Chen K. Anergic B cells: Precarious on-call warriors at the nexus of autoimmunity and false-flagged pathogens. Front Immunol (2015) 6:580. doi: 10.3389/fimmu.2015.00580 26635794 PMC4659919

[66] Gonzalez-Quintial R Mayeux JM Kono DH Theofilopoulos AN Pollard KM Baccala R. Silica exposure and chronic virus infection synergistically promote lupus-like systemic autoimmunity in mice with low genetic predisposition. Clin Immunol (2019) 205:75–82. doi: 10.1016/j.clim.2019.06.003 31175964 PMC6646094

[67] Lu M Munford R. LPS stimulates IgM production *in vivo* without help from non-B cells. Innate Immun (2016) 22(5):307–15. doi: 10.1177/1753425916644675 27189424

[68] Frasca D Romero M Diaz A Alter-Wolf S Ratliff M Landin AM. A molecular mechanism for TNF-alpha-mediated downregulation of b cell responses. J Immunol (2012) 188(1):279–86. doi: 10.4049/jimmunol.1003964 PMC3700394 22116831

[69] Janosevic D Myslinski J McCarthy TW Zollman A Syed F Xuei X. The orchestrated cellular and molecular responses of the kidney to endotoxin define a precise sepsis timeline. Elife (2021) 10:e62270. doi: 10.7554/eLife.62270 33448928 PMC7810465

[70] Fillatreau S. B cells and their cytokine activities implications in human diseases. Clin Immunol (2018) 186:26–31. doi: 10.1016/j.clim.2017.07.020 28736271 PMC5844600

