# Supplementary figures and images for: Autoreactive B cells recruited to lungs by silica exposure contribute to local autoantibody production in autoimmune-prone BXSB and B cell receptor transgenic mice
Source: Front Immunol. 2022 Aug 2;13:933360. doi: 10.3389/fimmu.2022.933360 (PMC9378786; doi:10.3389/fimmu.2022.933360)

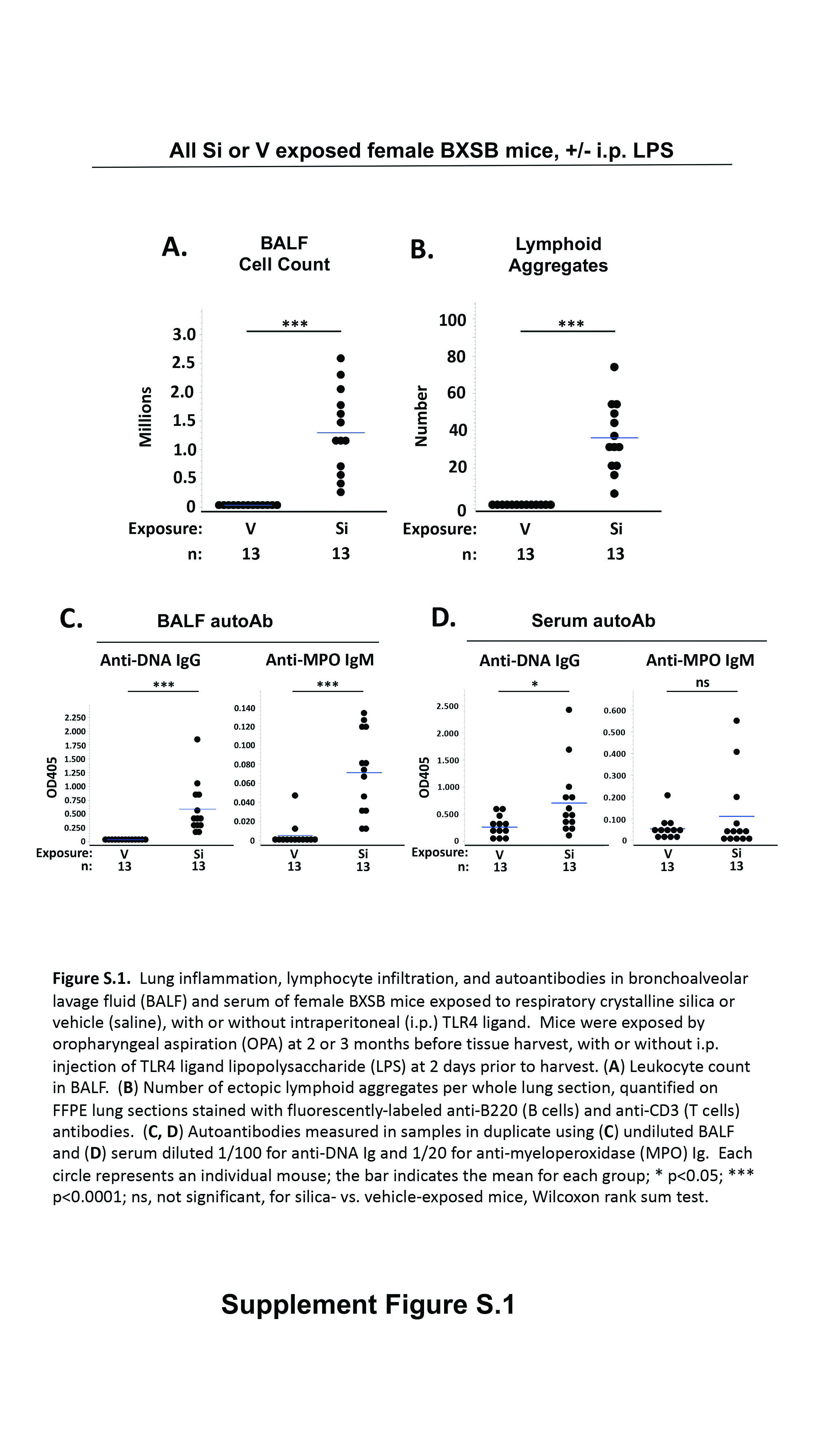

Supplement: Supplementary Figure 1 — Lung inflammation, lymphocyte infiltration, and autoantibodies in bronchoalveolar lavage fluid (BALF) and serum of female BXSB mice exposed to respiratory crystalline silica or vehicle (saline), with or without intraperitoneal (i.p.) TLR4 ligand. Mice were exposed by oropharyngeal aspiration (OPA) at 2 or 3 months before tissue harvest, with or without i.p. injection of TLR4 ligand lipopolysaccharide (LPS) at 2 days prior to harvest. (A) Leukocyte count in BALF. (B) Number of ectopic lymphoid aggregates per whole lung section, quantified on FFPE lung sections stained with fluorescently-labeled anti-B220 (B cells) and anti-CD3 (T cells) antibodies. (C, D) Autoantibodies measured in samples in duplicate using (C) undiluted BALF and (D) serum diluted 1/100 for anti-DNA Ig and 1/20 for anti-myeloperoxidase (MPO) Ig. Each circle represents an individual mouse; the bar indicates the mean for each group; *p < 0.05; ***p < 0.0001; ns, not significant, for silica- vs. vehicle-exposed mice, Wilcoxon rank sum test. [file Image_1.jpg]
